# Antibody Discovery *Ex Vivo* Accelerated by the LacO/LacI Regulatory Network

**DOI:** 10.1371/journal.pone.0036032

**Published:** 2012-04-27

**Authors:** Munehisa Yabuki, W. Jason Cummings, John B. Leppard, Robert M. Immormino, Christi L. Wood, Daniel S. Allison, Patrick W. Gray, Larry W. Tjoelker, Nancy Maizels

**Affiliations:** 1 Department of Immunology, University of Washington School of Medicine, Seattle, Washington, United States of America; 2 XORI Corporation, Seattle, Washington, United States of America; 3 Accelerator Corporation, Seattle, Washington, United States of America; 4 Department of Biochemistry, University of Washington School of Medicine, Seattle, Washington, United States of America; Aberystwyth University, United Kingdom

## Abstract

Monoclonal antibodies (mAbs) can be potent and highly specific therapeutics, diagnostics and research reagents. Nonetheless, mAb discovery using current *in vivo* or *in vitro* approaches can be costly and time-consuming, with no guarantee of success. We have established a platform for rapid discovery and optimization of mAbs *ex vivo*. This DTLacO platform derives from a chicken B cell line that has been engineered to enable rapid selection and seamless maturation of high affinity mAbs. We have validated the DTLacO platform by generation of high affinity and specific mAbs to five cell surface targets, the receptor tyrosine kinases VEGFR2 and TIE2, the glycoprotein TROP2, the small TNF receptor family member FN14, and the G protein-coupled receptor FZD10. mAb discovery is rapid and humanization is straightforward, establishing the utility of the DTLacO platform for identification of mAbs for therapeutic and other applications.

## Introduction

Monoclonal antibodies (mAbs) are well-established as therapeutics, diagnostics, and reagents for research, but their use is currently limited by the difficulties and costs associated with identifying mAbs with the required affinity and specificity. Many targets of interest are highly conserved proteins, and immune regulation limits antibodies that can be obtained from a physiological immune response. In addition, many key therapeutic targets are cell surface proteins, which present particular challenges to mAb development because their physiologically active conformations are not readily recapitulated by purified proteins or membrane preparations used for immunization to elicit specific antibodies. This includes some especially high value targets, such as cytokine receptors and G protein-coupled receptors.

Most current strategies for mAb discovery depend on *in vivo* and *in vitro* approaches. *In vivo* approaches depend on activation and selection of specific B cells by immunization, followed by generation of hybridomas [1], [2]. This process is costly and time-consuming, since extensive screening and, in many cases, subsequent steps including affinity maturation are required to obtain mAbs with desired properties. It is also limited by immune tolerance, making some antigens difficult or impossible to target. In addition, once a mAb has been identified there is not a straightforward path to further optimization of affinity or functionality. *In vitro* approaches rely on screening massive numbers of synthetic single-chain antibodies, typically displayed on phage [3], [4]. These antibodies are expressed by cloned genes that encode linked V_H_ and V_L_ regions derived from an immune repertoire, often from a convalescent individual [5], [6]. They can be further optimized by iterative PCR-based mutagenesis accompanied by selection *in vitro*, using high throughput approaches. However, success in the end depends on the quality of the starting libraries and their sources, and not all single-chain antibodies can be readily converted to natural antibodies for practical applications.

mAb discovery can also be carried out *ex vivo* in immortalized B cells. B cells display immunoglobulin (Ig) molecules on the cell surface, facilitating selection for antigen recognition. In some B cell lines, physiological pathways for Ig gene diversification remain active, enabling evolution of high affinity antibodies in culture. The chicken B cell line, DT40, has proven especially adaptable for such purposes [7], [8], [9]. DT40 derives from a bursal lymphoma, and cells constitutively diversify their V_H_ and V_L_ genes [10]. Ongoing diversification occurs by two pathways [11]. Most mutations are templated and arise as a result of gene conversion, with nonfunctional pseudo-V regions serving as donors for transfer of sequence to the rearranged and transcribed V gene. A small fraction of mutations are nontemplated, and arise as a result of somatic hypermutation, the mutagenic pathway that generates point mutations in Ig genes of antigen-activated human and murine B cells. DT40 cells proliferate rapidly, with an 8–10 hr doubling time (compared to 20–24 hr for human B cell lines), and are robust to experimental manipulations including magnetic-activated cell sorting (MACS), fluorescence-activated cell sorting (FACS) and single-cell cloning. Most importantly, DT40 cells support very efficient homologous gene targeting [12], so genomic regions can be replaced or modified at will.

Despite the considerable potential of DT40 cells for antibody evolution, their utility has thus far been limited in practice because — as in other transformed B cell lines — Ig gene diversification occurs at less than 1% the physiological rate. Several approaches have been used to accelerate diversification in DT40 cells. Disabling the homologous recombination pathway accelerates point mutagenesis, but cells thus engineered have lost the ability to diversify their Ig genes by gene conversion or to carry out gene targeting; and all mutations are nontemplated point mutations, like those generated during antigen-driven somatic hypermutation in humans or mice [7]. Treatment of cells with the histone deacetylase inhibitor, trichostatin A accelerates gene conversion [8], but does not promote point mutagenesis, limiting potential diversity. By transfecting DT40 cells with inducible transgenes expressing factors necessary for homologous repair, it is possible to toggle between diversification that generates templated or nontemplated mutations [9], but this does not significantly affect the rate of diversification.

We have now engineered DT40 cells to accelerate the rate of Ig gene diversification, without sacrificing the capacity for further genetic modification or the potential for both gene conversion and somatic hypermutation to contribute to V region mutagenesis. Distinct steps of engineering were carried out to generate the DTLacO platform, which enables rapid and seamless selection of high affinity mAbs *ex vivo* under control of the potent LacO/LacI regulatory network. We have demonstrated generation of high affinity mAbs against six targets, including the model antigen, streptavidin (SAv), and five cell surface antigens, the receptor tyrosine kinases VEGFR2 and TIE2, the glycoprotein TROP2, the TNF receptor family member FN14, and the G protein-coupled receptor FZD10. These results establish the power of the DTLacO platform for identification of mAbs for therapeutic and other applications.

## Results

### The DTLacO mAb discovery platform

The DTLacO platform for rapid mAb selection and optimization was developed by engineering DT40 cells to put diversification under control of the powerful *E. coli* LacO/LacI regulatory network. Regulation by LacO/LacI takes advantage of the high-affinity (k_D_ = 10^−14^ M) of lactose repressor (LacI) for lactose operator (LacO), as well as the sensitivity of the LacI/LacO interaction to the small molecule, IPTG. We initially generated a cell line, DT40 PolyLacO-λ_R_, in which polymerized lactose operator (“PolyLacO") had been upstream of the rearranged and expressed immunoglobulin λ light chain gene (Igλ_R_) by homologous gene targeting [13]. We showed that, in this line, the rate and outcome of diversification could be controlled by expression of distinct regulatory factors fused to LacI [13], [14], [15].

**Figure 1:**
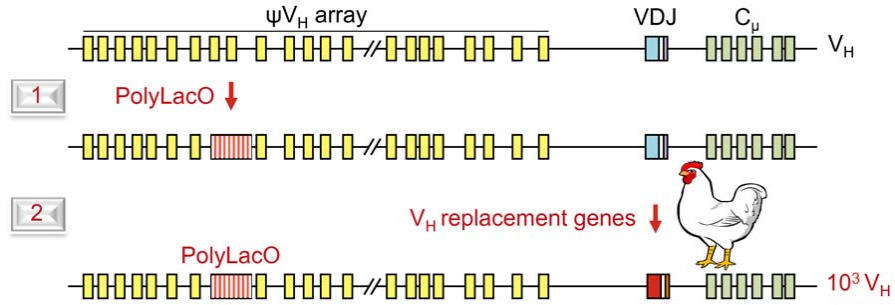
Two steps of engineering accelerate clonal diversification. Schematic diagram of the rearranged and expressed IgH locus, showing the variable (VDJ) region, the constant (C_μ_) region, and the upstream ψV_H_ array. IgH was first modified by insertion of PolyLacO within the ψV_H_ array in DT40 PolyLacO-λ_R_ cells, which carry PolyLacO targeted to the rearranged and expressed Igλ locus [13], [14], [15]. Next, this locus was further modified by substitution of the endogenous V_H_ (VDJ) region with V_H_ regions from a naive chick.

We then further modified these cells in two steps (Figure 1), both of which took advantage of the high efficiency of gene targeting in the parental chicken DT40 B cell line. First, we inserted the potent PolyLacO regulatory element upstream of the rearranged and expressed IgH gene in the DT40 PolyLacO-λ_R_ cell line, which carries PolyLacO at Igλ [13]. This created the DTLacO-1 population, which carries PolyLacO at both Igλ and IgH, upstream of the endogenous rearranged V_H_ (VDJ) and V_λ_ (VJ) regions. Next, we substituted a V_H_ library generated from chicken bursal B cells for the single endogenous V_H_ region. This expanded the initial V_H_ repertoire, and created the DTLacO-2 population.

### Synergistic acceleration of diversification by PolyLacO targeted to both Igλ and IgH

**Figure 2:**
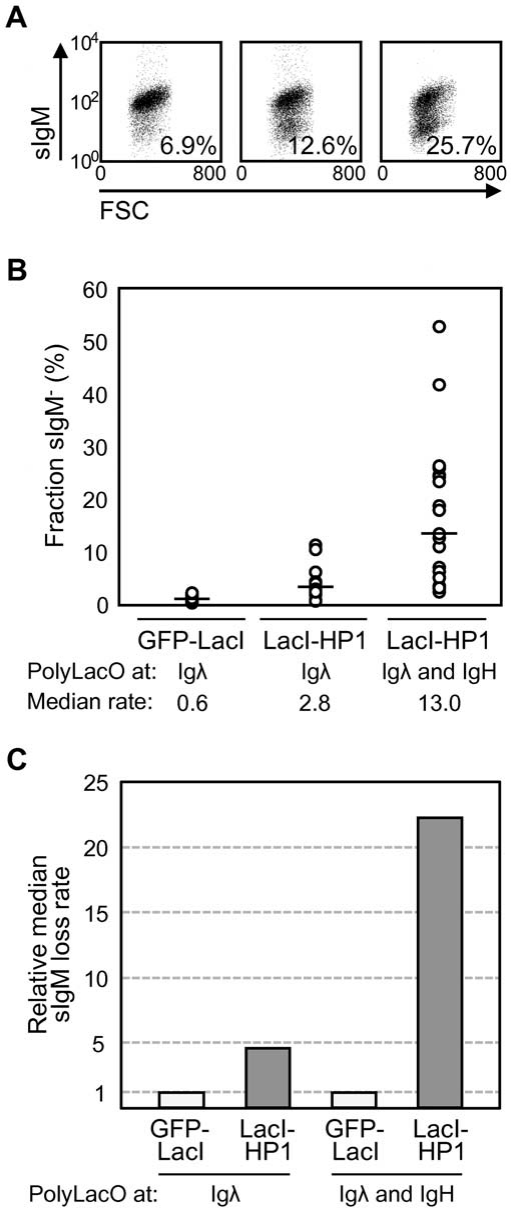
Clonal diversification rate accelerated in DTLacO cells. (A) sIgM loss assay of three representative clonal DTLacO LacI-HP1 transfectants. Fraction of sIgM^−^ cells in each culture indicated at lower right in each panel. (B) Summary of sIgM loss assays. Each open circle represents the percentage of sIgM^−^ cells in one clonal transfectant, analyzed 3 weeks post-transfection. Cells analyzed were: DT40 PolyLacO-λ_R_ GFP-LacI control transfectants (n = 27); DT40 PolyLacO-λ_R_ LacI-HP1 transfectants (n = 16) and DTLacO LacI-HP1 transfectants (n = 20). (C) Median sIgM loss of DT40 PolyLacO-λ_R_ LacI-HP1 and DTLacO LacI-HP1 transfectants relative to GFP-LacI control transfectants.

The presence of PolyLacO at both Igλ and IgH should accelerate diversification. We tested this by comparing diversification rates in DTLacO-1 cells, engineered to carry PolyLacO targeted to both the Igλ and the IgH genes, relative to DT40 PolyLacO-λ_R_ cells, which carry PolyLacO only at Igλ. Diversification rates of candidate engineered lines were determined by assaying the fraction of sIgM^−^ cells 3 weeks post-transfection with the LacI-HP1 regulatory factor. Representative candidates exhibited diversification rates of 6.9%, 12.6% and 25.7% (e.g. Fig. 2A), from 2.5- to 9.2-fold elevated relative to the 2.8% characteristic of the parental DT40 PolyLacO-λ_R_ LacI-HP1 line. Accelerated diversification was reconfirmed for one line by fluctuation assay of individual transfectants (Fig. 2B). Percentages of sIgM^−^ cells ranged from 2.5% to 52.5%, with a median of 13.0% (Fig. 2B), 4.6-fold higher than in DT40 PolyLacO-λ_R_ LacI-HP1 transfectants (2.8%), and 21.7-fold higher than in control DT40 PolyLacO-λ_R_ GFP-LacI cells (0.6%, comparable to the DT40 parental line [13]). Some individual clones exhibited sIgM loss considerably different than the median, as predicted because this fluctuation assay measures accumulated sIgM^−^ variants. Thus, targeting PolyLacO elements to both the heavy and light chain genes accelerated diversification nearly 22-fold relative to the DT40 parental cell line (Fig. 2C). Diversification was also accelerated upon transfection of these cells with other regulatory factors expressed as LacI fusions, including GFP-LacI-VP16 and E47-LacI (not shown).

### *Ex vivo* evolution of anti-streptavidin antibodies

**Figure 3:**
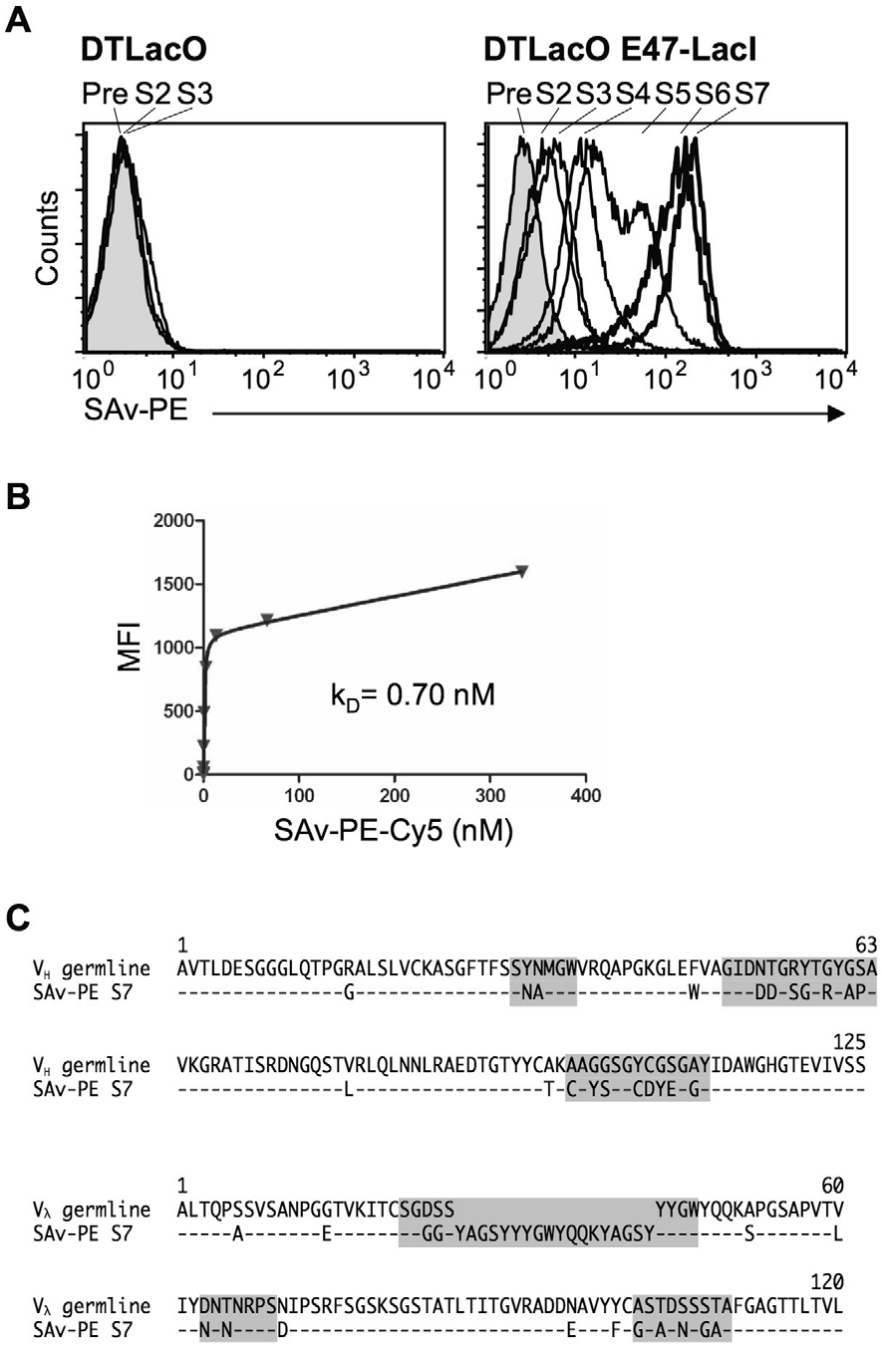
Rapid evolution of anti-SAv antibodies in DTLacO cells. (A) SAv binding profile of successive selected cell populations of DTLacO (left) or DTLacO E47-LacI (right) cells. Selection was carried out on average weekly. Cell numbers plotted relative to SAv-PE fluorescent signal. Populations at successive rounds of selection designated above peaks (S0–S7). Pre, populations prior to any sorting (gray fill). (B) Saturation binding kinetics of DTLacO E47-LacI S7 population. (C) Sequences of high affinity selected anti-SAv mAb compared to the germline [16], [17]. CDRs are identified by background shading. The D6 sequence was chosen as the germline D element for comparison [17]. Note the 18-residue insertion/duplication in CDR1 of V_λ_ of the anti-SAv mAb, recapitulating an insertion in light-chain CDR1 reported by others selecting anti-SAv mAbs from DT40 cells that had not undergone any genetic engineering [8].

To test the utility of DTLacO cells for *ex vivo* mAb evolution, we selected mAbs against the model antigen, streptavidin (SAv) [7], [8] from the DTLacO-1 population (Figure 1, Step 1). Cells were stably transfected with an E47-LacI expression construct, which encodes a fusion of LacI and the E47 isoform of the regulatory factor, E2A. E47 is a transcriptional regulator in some contexts, but at the Ig genes of DT40 cells it promotes diversification but not transcription [15]. A diversified population of 3×10^8^ DTLacO E47-LacI cells was enriched twice for binding to SAv-conjugated magnetic beads, then selected by successive rounds of FACS for binding to SAv-PE. The cell population exhibited increased affinity after each round of selection. A 30-fold shift was evident after the fifth round of selection and a 100-fold shift by the seventh round (S5 and S7, respectively; Figure 3A). The binding affinity of the S7 population for SAv-PE-Cy7 was measured by saturation binding kinetics. In this FACS-based method, cells are stained with increasing concentrations of antigen until equilibrium of bound and unbound antigen is established; the resulting mean fluorescence intensity (MFI) values are analyzed with Prism software (GraphPad); and the affinity at equilibrium (k_D_) is determined (Figure 3B). The apparent affinity was found to be 0.7 nM, after 7 rounds of selection, which compares favorably with 15–19 rounds of selection required for selection of antibodies of comparable affinity *ex vivo* using cultured human B cell lines [7]. The sequences of the V_H_ and V_λ_ regions were determined by PCR amplification from single cells, and compared to the germline [16], [17]. Strikingly, an 18 residue insertion/duplication was identified in CDR1 of V_λ_ (Figure 3C). An insertion in light chain CDR1 of anti-SAv mAbs has also been reported by others using DT40 cells that have not undergone any genetic engineering [8].

### Selection of high affinity mAbs that recognize conserved cell surface receptors

**Figure 4:**
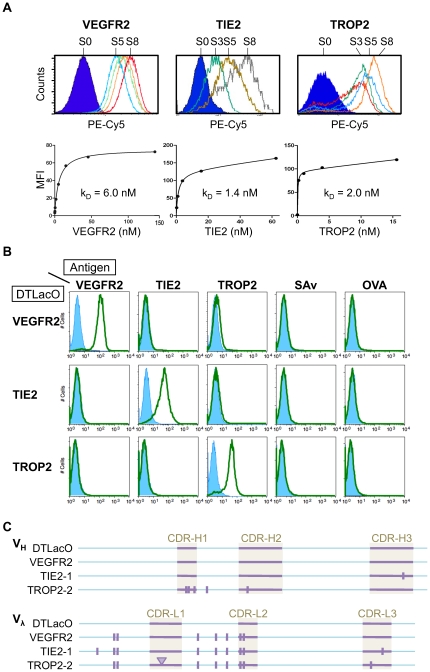
High affinity mAbs selected from DTLacO cells. (A) Above, binding profiles of successive DTLacO LacI-HP1 populations selected for recognition of cell surface receptors, VEGFR2, TIE2 and TROP2. Rounds of selection designated above peaks (S0–S8). Below, saturation binding kinetics, indicating apparent k_D_. (B) Specificity of selected DTLacO populations. FACS analysis of binding of cell populations selected for high affinity recognition of VEGFR2, TIE2 or TROP2 to recombinant VEGFR2, TIE2, TROP2, SAv or ovalbumin (OVA). Solid peaks represent the negative reference control (secondary antibody alone), and green lines represent staining with antigen. (C) Schematic alignment of V_H_ and V_λ_ regions of mAbs selected for binding to VEGFR2, TIE2 and TROP2. Thin horizontal blue lines represent chicken framework regions, thicker horizontal lavender lines against background shading identify CDRs, vertical bars indicate single residue differences relative to the most common DTLacO sequence, and triangle indicates insertion.

The DTLacO-1 cells stably expressing LacI-HP1 were selected to identify mAbs against three cell surface antigens of therapeutic interest: the receptor tyrosine kinases, VEGFR2 and TIE2, which play essential roles in physiologic and pathologic angiogenesis, most notably in cancer [18], [19]; and the glycoprotein, TROP2, which is overexpressed in numerous epithelial cancers [20]. The extracellular domains of these receptors are highly conserved, with the human and murine orthologs exhibiting 80%, 90%, and 83% identity, respectively. Each extracellular domain was expressed as recombinant protein fused to the human IgG1 Fc domain. DTLacO cells specific for each antigen were enriched from 1×10^9^ cells by initial selection on the antigen bound to magnetic beads and then for binding to the soluble antigen by FACS. Eight successive selected populations were characterized and shown to exhibit increased affinity at each selection step (Figure 4A, above). At the eighth selection step, analysis of saturation binding kinetics of the soluble antigens to their cognate DTLacO populations established apparent affinity values (k_D_) of 6.0, 1.4, and 2.0 nM, respectively (Figure 4A, below). Specificity of individual selected populations was tested by assaying binding to a panel of antigens (VEGFR2, TIE2, TROP2, SAv and ovalbumin). The selected DTLacO cells recognized only the cognate target, and were not cross-reactive (Figure 4B).

### CDR-targeted mutations characterize high affinity mAbs

Recombinant, chimeric chicken-human mAbs were generated by cloning the V_H_ and V_λ_ regions from the DTLacO cells that recognized VEGFR2, TIE2 or TROP2 into a construct for expression fused to human γ1 heavy- and λ light-chain constant regions. The chimeric mAbs preserved high affinity antigen recognition (data not shown), showing that the B cell receptor conferred high affinity binding by the selected cells. Sequence analysis of the cloned V_H_ and V_λ_ regions showed that mutations conferring high affinity and specificity mapped primarily to CDRs (Figure 4C). Both templated and nontemplated mutations were evident in the CDRs, although not all mutations in the heavy chain could be definitively assigned to a pathway, as the entire array of ψV_H_ segments has not yet been sequenced.

### Expanded V_H_ diversity further accelerates mAb selection

**Figure 5:**
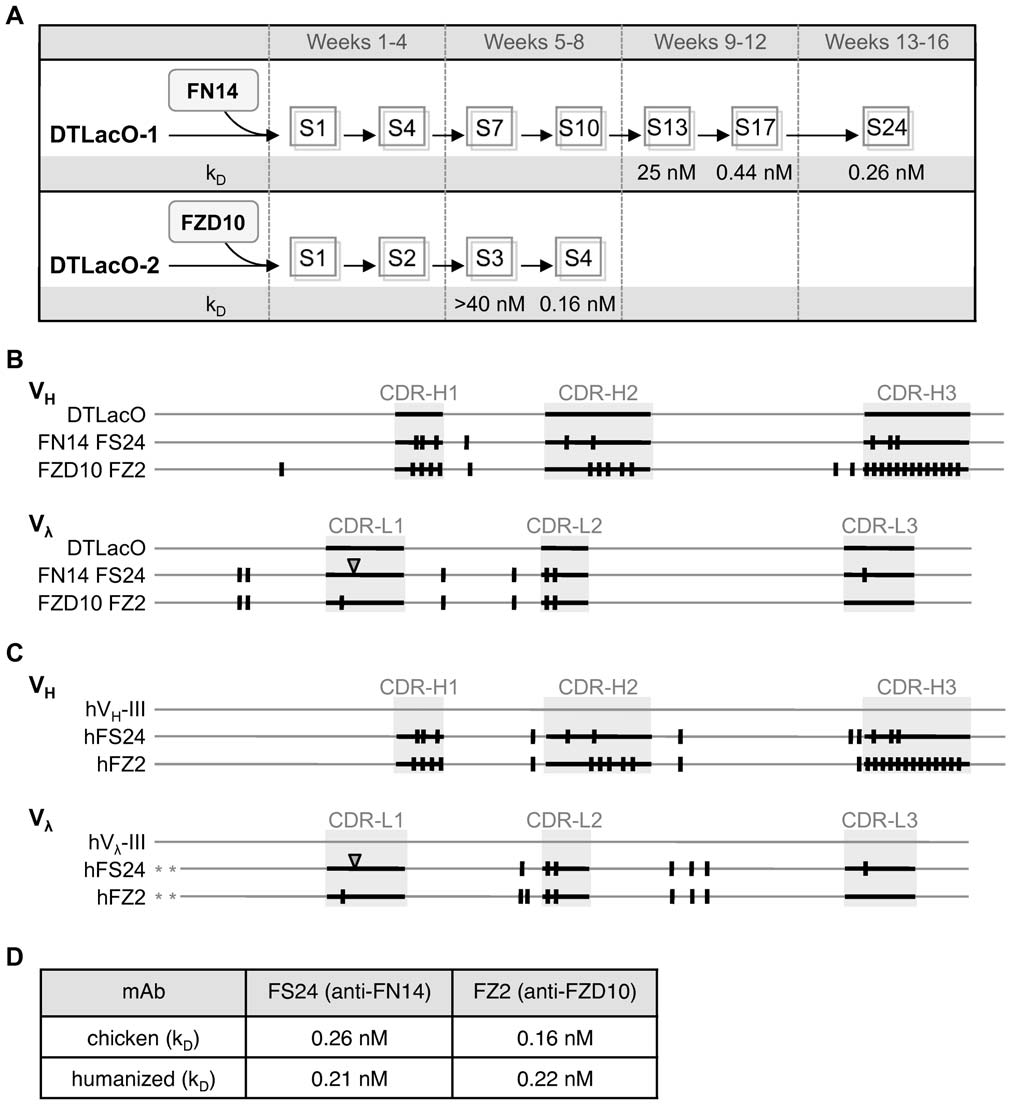
Selection and humanization of anti-FN14 and anti-FZD10 mAbs. (A) Schematic of time course of selection of anti-FN14 and anti-FZD10 mAbs, with selection steps indicated by S, and apparent affinities (k_D_) of recombinant chimeric mAbs shown below. (B) Schematic alignment of V_H_ and V_λ_ regions of mAbs selected for binding to FN14 and FZD10. Thin horizontal lines represent chicken framework regions, thicker horizontal lines against background shading identify CDRs, vertical bars indicate single residue differences relative to the most common DTLacO sequence, and triangle indicates insertion. (C) Antibody humanization. V_H_ and V_λ_ regions of humanized mAbs hFS24 and hFZ2 schematically aligned to the human V_H_-III or V_λ_-III consensus (top lines). Thin horizontal lines represent human framework regions; asterisks denote the two residues eliminated from the N-terminal of the light chain; vertical lines outside background shading identify Vernier zone residues preserved in humanized mAbs; other notations as in Panel B. (D) Apparent affinities (k_D_) of humanized and progenitor mAbs.

DTLacO cells expressing regulatory LacI-fusion factors, from either the initial population or the population in which the repertoire had been expanded by V_H_ replacement (DTLacO-1 and DTLacO-2, respectively; Figure 1), were the sources of mAbs recognizing two other antigens of therapeutic interest, the small TNF receptor family member, FN14 [21], and the G protein-coupled receptor, FZD10 [22]. Both proteins have highly conserved extracellular domains (92% and 94% identity, respectively, between human and mouse). An anti-FN14 mAb (FS24; Leppard et al., manuscript in preparation) was selected from the DTLacO-1 population and matured by LacI-HP1-driven diversification (Figure 5A). Subnanomolar affinity (k_D_ = 0.44 nM) was achieved after 17 rounds of selection over 12 weeks, and affinity improved only modestly in the course of 7 additional selections over the next 4 weeks (k_D_ = 0.26 nM). An anti-FZD10 mAb (FZ2; Cummings et al., manuscript in preparation) was selected from the DTLacO-2 population, with diversification accelerated by the tethered factor HIRA-GFP-LacI [14]. The population reached subnanomolar affinity after only four rounds of selection, over 8 weeks (Figure 5A). This mAb recognized its target with apparent affinity k_D_ = 0.16 nM. Sequence analysis of the cloned V_H_ and V_λ_ regions showed that mutations conferring high affinity and specificity mapped primarily to CDRs (Figure 5B; note that sequence analysis does not distinguish mutations in the mAb FZ2 V_H_ region that occurred as a result of V_H_ region swap and LacO/LacI-driven diversification.).

### Facile humanization of chicken antibodies

Antibodies selected in mice or other species are typically humanized for therapeutic applications [23]. The anti-FN14 and anti-FZD10 mAbs were chosen for humanization, as their high affinity and distinct heavy-chain CDRs offered a robust test of this key step in mAb development. Chicken V_H_ and V_λ_ regions are most closely related to human V_H_ subgroup III and V_λ_ subgroup III, respectively. These are well-established frameworks for humanization, and have been used previously to humanize mAbs elicited by immunization of chickens [24], [25]. The structure of a CDR is determined not only by the primary sequence of the CDR itself but also by a small number of nearby “Vernier zone" residues that contribute to shaping CDR structure [26]. Scaffolds for CDR grafting were generated by modifying human framework regions at the few positions necessary to achieve identity with the Vernier zone residues of the corresponding chicken V_H_ or V_λ_ region. The framework scaffolds thereby generated are 94–96% identical to human, making immunogenicity very unlikely. The first two N-terminal residues of light chains were also eliminated, as these residues lie proximal to CDR1 in mammalian antibodies and could in principle interfere with interaction with antigens. The CDRs of the chicken mAbs were then grafted to the modified scaffolds, to create the humanized V_H_ and V_λ_ regions (Figure 5C). Comparisons of apparent binding affinities of the humanized anti-FN14 and anti-FZD10 mAbs showed that humanization was achieved without loss of affinity (Figure 5D). This facile humanization contrasts to murine antibodies, which require considerable empirical optimization.

## Discussion

The DTLacO platform permits rapid *ex vivo* discovery of mAbs that recognize highly conserved targets. We have demonstrated the power of the DTLacO platform by generating specific and high affinity mAbs to five cell surface antigens of therapeutic interest, the receptor tyrosine kinases VEGFR2 and TIE2, the glycoprotein TROP2, the small TNF receptor family member FN14, and the G protein-coupled receptor FZD10. The highly conserved extracellular domains of these cell surface receptors are likely to make them difficult targets for *in vivo* mAb discovery, which is limited by immune tolerance. Time from initial selection to identification of a high-affinity mAb (<10 nM) was on the order of 4–8 weeks, and subnanomolar affinity was achieved in 8–12 weeks. This compares very favorably with other *ex vivo* or *in vivo* platforms for mAb discovery.

The DTLacO *ex vivo* mAb discovery platform provides several additional advantages relative to other mAb discovery approaches. The cells produce intact antibodies, which can immediately be tested for desired properties, whereas many *in vitro* approaches like phage-display system produce single-chain antibodies, which are frequently difficult to convert to active full-length mAbs due to aggregation or instability. The DTLacO cells diversify V regions using physiological pathways (somatic hypermutation and gene conversion), which target mutations mainly to CDRs, the subdomains of V regions that directly contact antigens. Furthermore, the cells proliferate rapidly and they are immortal, so at each step of selection the cell population provides not only a renewable source of antibodies (or V_H_ and V_L_ sequences for expression of recombinant antibodies), but also a starting point for further optimization.

The DTLacO platform is distinguished from other mAb discovery platforms based on DT40 cells [7], [8] by the ability to access both physiological diversification pathways, somatic hypermutation and gene conversion. DTLacO cells also retain the ability to carry out homologous gene targeting, which permits additional genetic engineering. We took advantage of this by substituting the endogenous V_H_ region with a V_H_ library, to create the DTLacO-2 population carrying an expanded V_H_ repertoire. The third heavy chain CDR, CDR-H3, includes the VDJ junction and is a major determinant for antigen recognition [27]. CDR-H3 diversity may have contributed to the rapid selection of a high affinity anti-FZD10 mAb from the DTLacO-2 population. It is also possible to swap human for chicken V regions (data not shown), which will permit optimizing affinity or functionality of mAbs discovered by other methods, as well as direct discovery of human therapeutic mAbs.

The chicken mAbs optimized in DTLacO cells proved to be readily humanized by CDR grafting into consensus human V_H_ subgroup III and V_λ_ subgroup III framework regions in which Vernier zone residues had been modified to preserve CDR structure. Humanization was carried out without loss of affinity, and achieved >94% identity to human within the framework regions. This is comparable to or better than many humanized murine mAbs now in the clinic, and makes immunogenicity very unlikely. The readiness with which the mAbs were humanized contrasts to antibodies discovered in mice or murine cells, which must undergo empirical optimization. V_H_ subgroup III and V_λ_ subgroup III framework regions are conserved among a number of vertebrates, raising the possibility that mAb frameworks could be modified for treatment of chronic illness in other species.

The rapid selection and humanization we have documented establish the utility of the DTLacO platform for therapeutic mAb discovery. Operationally, the platform is readily adaptable to high throughput approaches. This will facilitate application of the platform to discovery of mAbs for diagnostic and research applications, and make the platform especially well-suited for development of mAbs for personalized medicine.

## Materials and Methods

### Cell culture and gene targeting

DT40-derived cell lines ([13], [14], [15] and in this study) were maintained and transfected as previously described [28]. FreeStyle 293-F cells (Invitrogen) were maintained and transfected as specified by the manufacturer.

PolyLacO was targeted to the ψV_H_ array using the targeting construct, pPolyLacO-ψV_H_, designed to promote homologous recombination at the rearranged and expressed heavy chain allele of DT40 PolyLacO-λ_R_ cells. These cells had been previously engineered to carry the PolyLacO at the rearranged and expressed light chain allele [13], [14], [15]. To generate the pPolyLacO-ψV_H_ targeting construct, 2.8- and 4.2-kb homology arms were obtained from ψV_H_ array fragments amplified from DT40 genomic DNA using primers 5′-GGGGTCTCTATGGGGTCTAAGCGTGGCC-3′ and 5′-GGCCGATTCTTTTCTCATGAGATCCCTCCAGAAG-3′ or 5′-TTCCCCACAACCAGGCCATGCGCCTCCTTG-3′ and 5′-CCTGCAGACACCCAGAGGAGGGCTCAGC-3′. These two homology arms were subcloned into pBluescript II KS(+) (Stratagene), adjacent to a blasticidin-resistance gene to enable selection of stable transfectants following about 10 days growth in 20 µg/ml concentration of blasticidin (Invitrogen). The PolyLacO regulatory element [29], which consists of approximately 100 repeats of a 20-mer lactose operator (LacO), and was a kind gift of A.S. Belmont (U. Illinois, Urbana), was cloned between the homology arms. The construct was verified by restriction analyses and partial sequencing, and propagated in recombination-deficient *E. coli* strains Stbl2 (Invitrogen) to maintain repeat stability. Targeting was carried out essentially as previously described [15]. DT40 PolyLacO-λ_R_ cells were transfected, and stable transfectants were screened by genomic PCR and Southern blotting to identify homologous integrants.

The V_H_ (VDJ) region repertoire of DTLacO cells was expanded in two steps of gene targeting, both of which relied on the targeting vector, pVDJ3. To generate pVDJ3, 2.2- and 1.8-kb homology arms were amplified from DT40 genomic DNA using primers 5′-TGAATGCTTTGTTAGCCCTAATTAGGGATTGAATTGAGAG-3′ and 5′-CCGTGAGACCCCCCGTTGACC-3′ or 5′-GCCCGACCGAAGTCATCGTCTCCTCCGGTG-3′ and 5′-TTTGCCTTCAAATCACCCTA-3′, respectively, and fused to the leader-VDJ region and cloned into pBluescript II KS(+). The pVDJ3-GFP targeting construct derivative was generated by replacing the leader-VDJ region with a GFP expression cassette [30]. The pVDJ3-Bin1 targeting construct pool was generated by inserting a library of V_H_ regions into the XcmI-PshAI site of pVDJ3. Those sequences had been amplified from the bursa of a 2 month-old White Leghorn chick using PCR primers 5′-GGGTCTGCGGGCTCTATGGGG-3′ and 5′-ATCGCCGCGGCAATTTTGGGG-3′. Expansion of the repertoire was accomplished by first replacing the endogenous VDJ region in DTLacO cells with a GFP expression cassette using pVDJ3-GFP; and next replacing GFP with the pVDJ3-Bin1 targeting construct pool, producing sIgM^+^ cells.

Transfections for heavy chain targeting were carried out using a Nucleofector (program B-023; Lonza). sIgM^+^ cells were collected by MACS and then FACS. Briefly, following 2 days posttransfection, cells were washed in PBS containing 1% BSA (Sigma), and sIgM^+^ cells enriched by binding to protein G Dynabeads (Dynal) coupled to anti-chicken IgM (Southern Biotech) according to manufacturers' directions. After two days culture, sIgM^+^/GFP^−^ cells were sorted using a FACSAria (BD Biosciences), generating the DTLacO-2 population.

### Quantitation of diversification rates

Diversification rates were quantified using the sIgM loss assay, which measures the fraction of cells that have lost expression of IgM on the cell surface due to diversification events [28], [31], [32]. In brief, panels of approximately 20 independent transfectants were expanded for 3 weeks, then cells (∼1×10^6^) from each panel member were stained with R-PE conjugated anti-chicken IgM (1∶200; Southern Biotech), and analyzed on a FACScan with CellQuest software (BD Biosciences). Cells with fluorescence intensity less than one-eighth the median of the sIgM^+^ peak were scored as sIgM^−^ [31], [33].

### V region sequence analysis

V-region PCR and sequence analysis were performed essentially as described [13], [28], using primers 5′-CAGGAGCTCGCGGGGCCGTCACTGATTGCCG-3′ and 5′-GCGCAAGCTTCCCCAGCCTGCCGCCAAGTCCAAG-3′ for amplification of the rearranged V_λ_ regions and primers 5′-GGGTCTGCGGGCTCTATGGGG-3′ and 5′-ATCGCCGCGGCAATTTTGGGG-3′ for amplification of the rearranged V_H_ regions When necessary, semi-nested PCR was carried out using a second-round primer 5′-TCACTGATTGCCGTTTTCTCCCCTCTCTCC-3′ for the V_λ_ regions or 5′-GGTCAACGGGGGGTCTCACGG-3′ for the V_H_ regions. PCR products were purified with QIAquick PCR purification kit (Qiagen) and sequenced directly.

### Antigens and selection for antigen binding

Initial selections were performed by binding diversified DTLacO populations to magnetic beads complexed with antigens, and subsequent selections by FACS using fluorescence-labeled soluble antigens, following procedures previously described [7], [8] with minor modifications. In some cases, pre-clearing of non-specific DTLacO cells was carried out using unbound beads to efficiently enrich positive populations. SAv Dynabeads M-280 (Dynal) and SAv-PE (Southern Biotech) were used to select cells that recognized SAv. Selection of cells that recognized human cell surface proteins used recombinant human chimeric proteins, expressed as fusions with human IgG1 Fc (R&D Systems), including the extracellular domain of VEGFR2 (residues 20–764; Cat. no. 357-KD), TIE2 (residues 23–745; Cat. no. 313-TI), TROP2 (residues 88–274; Cat. no. 650-T2), FN14 (residues 28–79; Cat. no. 1199-TW) or FZD10 (residues 21–161; Cat. no. 3459-FZ). Chimeric proteins were bound to protein G Dynabeads (Dynal) using manufacturers' recommended conditions for MACS method, and detected with PE-Cy5-labeled anti-human IgG Fc (Southern Biotech; 1∶200) for FACS method. Antigens for selection were used at concentrations of 10 µg/ml; selections were carried out on >10^8^ cells at a bead∶cell ratio ranging from 3∶1 to 1∶1.

### Binding and affinity assays

Saturation binding kinetics were determined by staining cells with various concentrations of fluorescent-labeled soluble antigens, and apparent affinities (k_D_) were calculated by nonlinear regression using GraphPad Prism software. To test binding of mAbs to the cell surface antigens, recombinant chimeric chicken-human mAbs were generated by cloning PCR-amplified V_H_ and V_λ_ segments in frame into pcDNA3.1 (Invitrogen) derivatives, pcDNA3.HG1 and pcDNA3.HLam, carrying the human γ1 and λ constant regions, respectively. The expression plasmids were cotransfected transiently into FreeStyle 293-F cells (Invitrogen) according to the manufacturer's instruction. After 2–4 days culture, secreted antibodies were purified from supernatants by protein A chromatography (MabSelect SuRe; GE Healthcare) and, if necessary, concentrated by Ultracel ultrafiltration (Millipore). Target cells were generated by transient transfection of 293-F cells with antigen expression constructs (GeneCopoeia).

### Antibody humanization

To humanize the chicken mAbs, CDRs of anti-FN14 and anti-FZD10 mAbs were grafted into human frameworks V_H_ subgroup III and V_λ_ subgroup III [23]. Vernier zone residues [26] were modified by Quikchange site-directed mutagenesis (Agilent Technologies). The humanized V_H_ and V_λ_ regions were then transferred into pcDNA3.1-derived human IgG1 or Igλ expression constructs, and mAbs were expressed and purified using protein A as above.
